# Adhesion preference of the sticky bacterium *Acinetobacter* sp. Tol 5

**DOI:** 10.3389/fbioe.2024.1342418

**Published:** 2024-02-05

**Authors:** Shogo Yoshimoto, Satoshi Ishii, Ayane Kawashiri, Taishi Matsushita, Dirk Linke, Stephan Göttig, Volkhard A. J. Kempf, Madoka Takai, Katsutoshi Hori

**Affiliations:** ^1^ Department of Biomolecular Engineering, Graduate School of Engineering, Nagoya University, Nagoya, Aichi, Japan; ^2^ Department of Bioengineering, Graduate School of Engineering, The University of Tokyo, Tokyo, Japan; ^3^ Department of Biosciences, University of Oslo, Oslo, Norway; ^4^ Institute for Medical Microbiology and Infection Control, University Hospital, Goethe University, Frankfurt, Germany

**Keywords:** bacteria, adhesion, antiadhesive materials, autotransporters, *Acinetobacter*

## Abstract

Gram-negative bacterium *Acinetobacter* sp. Tol 5 exhibits high adhesiveness to various surfaces of general materials, from hydrophobic plastics to hydrophilic glass and metals, via AtaA, an *Acinetobacter* trimeric autotransporter adhesin Although the adhesion of Tol 5 is nonspecific, Tol 5 cells may have prefer materials for adhesion. Here, we examined the adhesion of Tol 5 and other bacteria expressing different TAAs to various materials, including antiadhesive surfaces. The results highlighted the stickiness of Tol 5 through the action of AtaA, which enabled Tol 5 cells to adhere even to antiadhesive materials, including polytetrafluoroethylene with a low surface free energy, a hydrophilic polymer brush with steric hindrance, and mica with an ultrasmooth surface. Single-cell force spectroscopy as an atomic force microscopy technique revealed the strong cell adhesion force of Tol 5 to these antiadhesive materials. Nevertheless, Tol 5 cells showed a weak adhesion force toward a zwitterionic 2-methacryloyloxyethyl-phosphorylcholine (MPC) polymer-coated surface. Dynamic flow chamber experiments revealed that Tol 5 cells, once attached to the MPC polymer-coated surface, were exfoliated by weak shear stress. The underlying adhesive mechanism was presumed to involve exchangeable, weakly bound water molecules. Our results will contribute to the understanding and control of cell adhesion of Tol 5 for immobilized bioprocess applications and other TAA-expressing pathogenic bacteria of medical importance.

## 1 Introduction

Bacterial adhesion causes a variety of problems such as infectious diseases, metal corrosion, and pathogen contamination of medical and food processing equipment, but it can be beneficial in bioreactors for wastewater treatment and off-gas treatment, degradation of pollutants in aqueous and soil environments, and chemical production using immobilized bacteria (Hori and Matsumoto, 2010). *Acinetobacter* sp. Tol 5, a toluene-degrading bacterium isolated from a biofiltration system, exhibits autoagglutination and high adhesiveness to solid surfaces (Hori et al., 2001; Ishikawa et al., 2012b). Tol 5 cells quickly adhere to various material surfaces, from hydrophobic plastics to hydrophilic glass and metals, independent of biofilm formation (Ishikawa et al., 2012b). Atomic force microscopy (AFM) revealed that the adhesion force of Tol 5 to a sharp silicon probe was near 2 nN, which was one to two orders of magnitude stronger than that of other highly adhesive bacteria (Ishii et al., 2022). This characteristic nonspecific adhesiveness of Tol 5 cells is mediated by a single protein, AtaA (Ishikawa et al., 2012a; Yoshimoto et al., 2016; Noba et al., 2019), a member of the trimeric autotransporter adhesin (TAA) family (Lyskowski et al., 2011). TAAs are outer membrane proteins of Gram-negative bacteria and have been well studied as virulence factors because they enable binding to biotic molecules of mammalian host cells and, in some cases, to various abiotic surfaces (Linke et al., 2006; Muller et al., 2011). Although they vary in sequence length from several hundred to several thousand amino acids, they have a common structure that includes an N-terminal passenger domain (PSD), which is secreted onto the cell surface and is responsible for its function, and a C-terminal transmembrane domain, which anchors the PSD in the outer membrane (Linke et al., 2006; Hartmann et al., 2012). The AtaA of *Acinetobacter* sp. Tol 5 is one of the largest TAAs known to date. It consists of 3,630 amino acids per monomer and shares common structural features with other TAAs (Ishikawa et al., 2012a; Koiwai et al., 2016), and its N-terminal head domain is essential for the adhesive function of AtaA (Yoshimoto et al., 2023).

Bacterial immobilization is an important and common way to use whole-cell biocatalysts efficiently, and various immobilization methods, such as physical adsorption, gel entrapment, and biofilm immobilization, have been developed and improved (Cassidy et al., 1996; Halan et al., 2012; Es et al., 2015). However, these conventional methods have practical limitations, such as limited mass transfer in the inner part of a gel, gel fragility, cell leakage from the support matrix, and adverse effects on cell viability and catalytic activity (Cassidy et al., 1996; Junter and Jouenne, 2004; Carballeira et al., 2009). The adhesive feature of AtaA can be conferred to other non-adhesive and non-agglutinating Gram-negative bacteria by transformation with *ataA* gene (Ishikawa et al., 2012a). We have previously developed a new method for bacterial cell immobilization using AtaA (Ishikawa et al., 2014; Yoshimoto et al., 2023). Large amounts of growing, resting, and even lyophilized transformant cells can be quickly and firmly immobilized onto any material surfaces selected according to the application (Hori et al., 2015). Cells immobilized directly on surfaces through AtaA are not embedded in extracellular polymeric substances with mass transfer limitations, show enhanced tolerance, increase chemical reaction rates, and can be repeatedly used in reactions without inactivation (Ishikawa et al., 2014). For efficient and stable immobilization of bacterial cells on carriers, it is important to know their adhesion preference. Although the adhesion of Tol 5 is nonspecific, Tol 5 cells may have prefer materials for adhesion.

While bacterial adhesion can be used for the immobilization, it is also an initial step of both infection by pathogens and the biofouling of equipment (Hall-Stoodley et al., 2004; Hori and Matsumoto, 2010; Banerjee et al., 2011; Berne et al., 2018). Therefore, to prevent the undesired bacterial adhesion, various antiadhesive surfaces, such as fluoropolymers, polymer brushes, highly hydrophilic zwitterionic polymers, and ultrasmooth or nanopatterned surfaces, have been developed and characterized (Pereni et al., 2006; Cheng et al., 2007; Sin et al., 2014; Zeng et al., 2014; Hasan and Chatterjee, 2015; Lu et al., 2017; Elbourne et al., 2019; Ivanova et al., 2020). Studying the adhesion of Tol 5 to these antiadhesive surfaces may be a good way to understand the adhesion preferences of Tol 5.

In this study, we investigated the interaction of Tol 5 and several other TAA-expressing bacterial strains with various materials, including antiadhesive surfaces that have different repelling mechanisms.

## 2 Materials and methods

### 2.1 Materials

A polyurethane foam support (1 cm^3^ cube; CFH-30) was obtained from Inoac Corporation (Aichi, Japan). Polystyrene plates (PS2035-1), glass plates (FF-001), stainless steel plates (SUS430 grade; EA441WA-21), PTFE plates (J1-537-01), mica disks (V-1 grade), and square glass tubes (VitroTubes, 8100) were purchased from Hikari Co., Ltd. (Osaka, Japan), Matsunami Glass Ind., Ltd. (Osaka, Japan), ESCO Co., Ltd. (Osaka, Japan), AS ONE Corp. (Osaka, Japan), TED PELLA, Inc. (CA, United States), and Vitrocom (Mountain Lakes, NJ), respectively. A poly (mOEGMA) brush surface was prepared on glass plates as described previously (Azuma et al., 2017). The MPC polymer-coated surface for the adhesion assays was prepared as described below. A glass plate was dipped into 2% MPC polymer solution (Lipidure-CM5206, a copolymer of MPC and butyl methacrylate; NOF Corp., Tokyo, Japan) dissolved in ethanol and shaken at 70 rpm for 3 min. After shaking, the glass plate was rinsed in pure water and dried at 60°C for 3 h. The MPC polymer-coated glass tube was prepared as described below using the flow chamber system. A solution of 2% MPC polymer dissolved in ethanol was flushed through a square glass tube (50 mm in length and 1 mm in every internal dimension) at 10 cm/min for 3 min, and then pure water was flushed through at 50 cm/min for 2 min. After passing air through at 50 cm/min for 2 min, the glass tube was removed from the flow chamber system and dried at 60°C for 3 h.

AFM colloidal probes modified with silica (CP-PNPL-SIO-A), polystyrene (CP-PNPL-PS-A), and gold (CP-PNPL-Au-A) colloids 2 μm in diameter were purchased from NanoAndMore (Wetzlar, Germany). A poly (mOEGMA) brush-modified colloidal probe was prepared by grafting the poly (mOEGMA) brush onto the silica-colloidal probe as described previously (Azuma et al., 2017). An MPC polymer-coated colloidal probe was prepared by immersing the silica-colloidal probe in 0.2% MPC polymer solution and incubated for 1 h at room temperature. After incubation, the MPC polymer-coated colloidal probe was rinsed in pure water.

Microwells used in the adhesion assays with various material surfaces were constructed in our laboratory as follows: Four sheets of vinyl electrical tape (ASKUL Corporation, Tokyo, Japan) were stacked, and holes with a diameter of 6 mm were punched into the stacked tape. The punched tape was placed on a material plate as shown in Supplementary Figure S1.

Prior to use, glass was washed with piranha solution (H_2_SO_4_:30% H_2_O_2_ = 3:1) followed by rinsing with pure water. Mica was cleaved with scotch tape. The MPC polymer-coated surface was rinsed with pure water, and the other surfaces were rinsed with pure ethanol.

The static contact angles (SCAs) of air bubbles in water on each material surface were measured with a contact angle meter (CA-W, Kyowa Interface Science Co., Tokyo, Japan) at room temperature and are listed in Supplementary Table S1. Material substrates were immersed in water, and 10 μL of air bubbles were placed on the substrates.

### 2.2 Bacterial strains and culture conditions

The bacterial strains used in this study are listed in Supplementary Table S2. These bacterial strains were grown as previously described (Ishikawa et al., 2012a; Lu et al., 2013). *Acinetobacter* strains were grown in Luria-Bertani (LB) medium at 28°C for 8 h. An overnight culture of *Yersinia enterocolitica* strains grown in LB medium at 28°C was used to inoculate the LB medium at a 1:100 dilution, and the medium was incubated at 37°C for 6 h to induce *yadA* expression (El Tahir and Skurnik, 2001). *B. henselae* strains were grown for 5 days on Columbia agar supplemented with 5% sheep blood at 37°C in a humidified atmosphere with 5% CO_2_. The expression of trimeric autotransporter adhesins in each strain was confirmed by Western blotting using anti-AtaA_699–1014_ antiserum, anti-BadA antibodies (Riess et al., 2004), or anti-YadA antibodies (sc-22472; Santa Cruz Biotechnology, Inc., Dallas, TX, United States).

### 2.3 Polyurethane foam support adhesion assay

The polyurethane foam support adhesion assay was performed as previously described (Hori et al., 2015), with slight modifications. Bacterial cells were suspended in BS-N buffer (34.5 mM Na_2_HPO_4_, 14.7 mM KH_2_PO_4_, 15.5 mM K_2_SO_4_, pH 7.2), and the optical density of the cell suspension at 660 nm (OD_660_) was adjusted to 1.0. Four pieces of the polyurethane foam support were placed into 20 mL of the cell suspension and shaken at 115 rpm at 28°C. After a 30-min incubation with shaking, the transparency of the cell suspension was observed and photographed by a digital camera.

### 2.4 Microwell adhesion assay

Bacterial cells were suspended in BS-N buffer, and the OD_660_ was adjusted to 0.5. The cell suspensions (50 μL each) were placed into a microwell with the materials and incubated at 28°C for 10 min. When the antiadhesive surfaces were used, the incubation time was extended to 30 min and 2 h. The cell suspensions were removed after incubation using a pipet, and the wells were washed for 10 s by shaking in BS-N buffer at 70 rpm. Cells adhering to the well were stained with 50 μL of 0.1% crystal violet solution for 15 min and washed for 10 s by shaking in BS-N buffer at 70 rpm. Finally, the stain was eluted with 200 μL of 70% ethanol, and the absorbance of the elution at 590 nm (A_590_) was measured with a microplate reader (ARVO X3; PerkinElmer, Inc., MA, United States).

### 2.5 Single-cell force spectroscopy

Bacterial cells were immobilized on a glass bottom dish (FluoroDish, FD5040-100; World Precision Ins., Sarasota, FL, United States) as previously described (Ishii et al., 2022). Single-cell force spectroscopy was performed using a NanoWizard 3 BioScience AFM system (JPK Ins., Berlin, Germany) in contact mode at room temperature in BS-N buffer. For measurement, colloidal probes with a diameter of 2 μm and spring constants of 0.06–0.09 N/m were used. The spring constants of the cantilevers were measured using the thermal noise method. The parameters used in the measurement were as follows: Z-length: 3 μm; applied force: 0.2 nN; speed: 1 μm/s; cell surface dwell time: 0.1 s; and sample rate: 5,000 Hz.

### 2.6 Flow chamber system

A rectangular flow chamber system, which we have previously reported (Furuichi et al., 2018), was used with slight modifications: a syringe pump (Legato 200; KD Scientific, Holliston, MA) was directly connected to a square glass tube 50 mm in length and 1 mm in every internal dimension without using a three-way stopcock valve. Tol 5 cells were suspended in BS-N buffer at an OD_660_ of 0.2, and the suspension was subjected to sonication to break up the cell clumps. The glass tube with or without an MPC polymer coating was filled with the cell suspension and incubated for 10 min at room temperature. The suspension was replaced with fresh BS-N buffer by flowing slowly at 1 cm/min for 35 min, and the fluid velocity was increased stepwise. Live images of the behavior of Tol 5 cells during their adhesion and detachment to/from the inner surface of the glass tube were recorded under a digital microscope (VHX-200; Keyence, Osaka, Japan). Wall shear stress (τ) was calculated using Eq. 1, as previously described (Furuichi et al., 2018):

$$\tau =\frac{8\mu U}{D}$$ (1)

where *μ*, *U*, and *D* are the fluid viscosity, fluid velocity, and diameter of the glass tube, respectively. The equivalent diameter (*De*) was used for the value of *D* in Eq. 1, and was calculated according to Eq. 2:

$$\text{De}=\frac{4\text{A}}{\text{P}}$$ (2)

where *A* and *P* are the sectional area and wetted perimeter, respectively.

## 3 Results and discussion

### 3.1 Comparison of adhesiveness among bacterial strains exhibiting different TAAs

**FIGURE 1:**
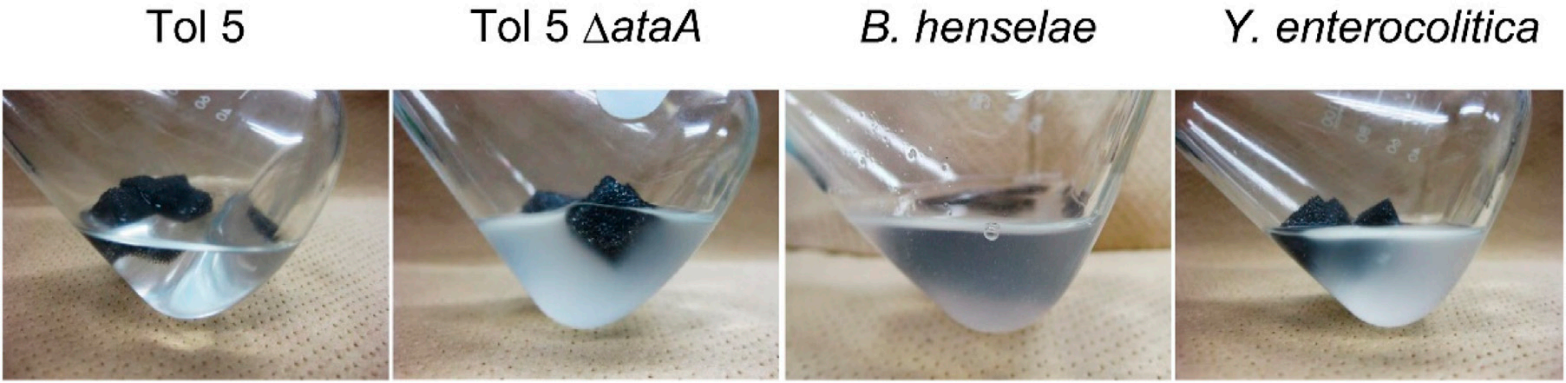
Adhesion of bacterial cells to a polyurethane surface. Each panel shows the bacterial cell suspension after shaking for 30 min with a polyurethane foam support.

First, we compared the adhesiveness of Tol 5 and its Δ*ataA* mutant with that of *Y. enterocolitica* WA-314 and *B. henselae* Marseille by shaking each cell suspension in the presence of a polyurethane support for 30 min. These Gram-negative bacteria have also been reported to adhere to abiotic surfaces through the peritrichate fibers of their TAAs (Muller et al., 2011), YadA and BadA, respectively. YadA is a well-studied short TAA consisting of 422 amino acids (GenBank: CBW54734.1). BadA (3,973 amino acids) (GenBank: MK993576.1) is slightly larger than AtaA, and it has also been reported to be more adhesive than other TAAs. It is worth noting that all of these TAAs show sequence and length variations among different strains and isolates (Muhlenkamp et al., 2017; Thibau et al., 2022). The expression of these TAAs was confirmed by Western blotting (see Supplementary Figure S2). In adhesion assays in shaking flasks with a polyurethane support, Tol 5 cells expressing AtaA showed overwhelmingly high adhesiveness compared with that of cells expressing other TAAs (Figure 1). Most of the Tol 5 cells adhered to the polyurethane support during shaking, and consequently, the Tol 5 cell suspension appeared clear. In contrast, the cell suspensions of *Y. enterocolitica*, *B. henselae,* and the Tol 5 Δ*ataA* mutant remained cloudy, which indicated that many of the cells did not adhere to the polyurethane support.

**FIGURE 2:**
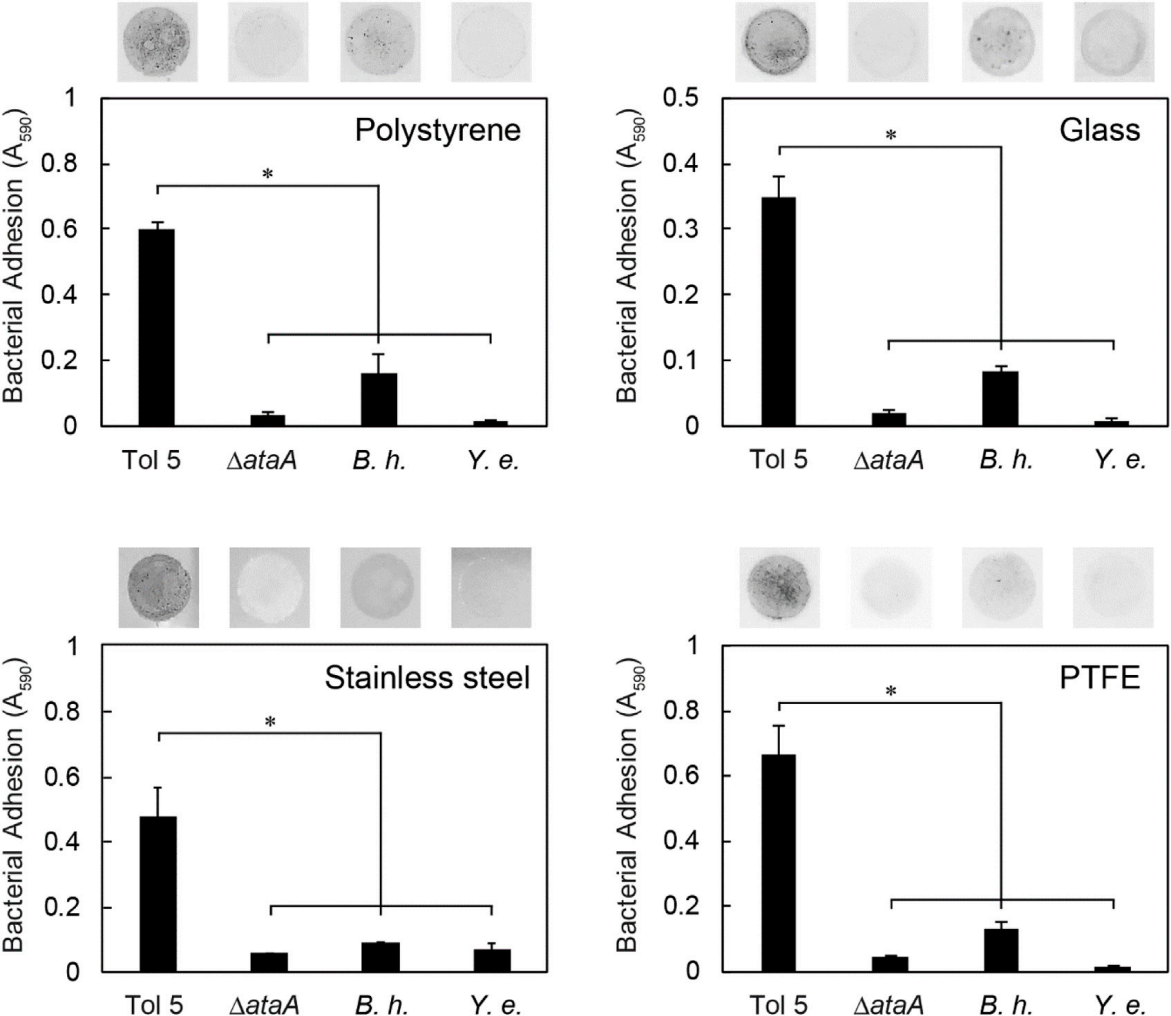
Instant adhesion of bacterial cells to various materials. Adhesion of *Acinetobacter* sp. Tol 5, Tol 5 Δ*ataA*, *Bartonella henselae* (*B. h.*), and *Yersinia enterocolitica* (*Y. e.*) to polystyrene, glass, stainless steel, and PTFE was assessed by microwell adhesion assays after 10 min of incubation. Data are expressed as the means ± SEMs (*n* = 3). Significant differences are indicated by an asterisk (Student’s t-test, *p* < 0.05). The upper photographs show the adhered cells on the material surfaces after staining.

Next, we measured the adhesion of bacterial cells expressing TAAs to the surface made from various materials (Table 1) using self-made microwells (Supplementary Figure S1). Cell suspensions were incubated on polystyrene (PS), glass, stainless steel, and polytetrafluoroethylene (PTFE, known as Teflon) surfaces for only 10 min. Nonadhering cells were removed by washing with fresh medium, and the instantly adhered cells on the material surface were quantified by crystal violet staining. Even in this short time, Tol 5 adhered not only to PS, glass, and stainless steel but also to PTFE, which has antiadhesive properties derived from its low surface energy (Figure 2) (Pereni et al., 2006). In contrast, the Tol 5 Δ*ataA* mutant and *Y. enterocolitica* hardly adhered to any of the material surfaces. Although *B. henselae* showed measurable adhesiveness, the amount of adhered cells was much smaller than that of Tol 5. These results quantitatively demonstrated that Tol 5 cells exhibit markedly higher adhesiveness to various material surfaces through the action of AtaA than bacterial cells expressing other TAAs.

**TABLE 1:** Surface materials used in this study.

Property	Microwell material	AFM probe
Hydrophobic	Polystyrene	Polystyrene
Hydrophilic	Glass	SiO_2_
Metal	Stainless steel	Gold
Low surface energy	PTFE/Teflon	–^a^
Atomically flat	Mica	–^a^
Strong hydrophilic/nonionic, steric hindrance	poly (mOEGMA) brush-coated glass	poly (mOEGMA) brush-coated SiO_2_
Strong hydrophilic/high free water fraction/zwitterionic	MPC polymer-coated glass	MPC polymer-coated SiO_2_

^a^ PTFE, and mica were not able to be used as AFM probes.

### 3.2 Adhesion of bacterial cells to antiadhesive surfaces

**FIGURE 3:**
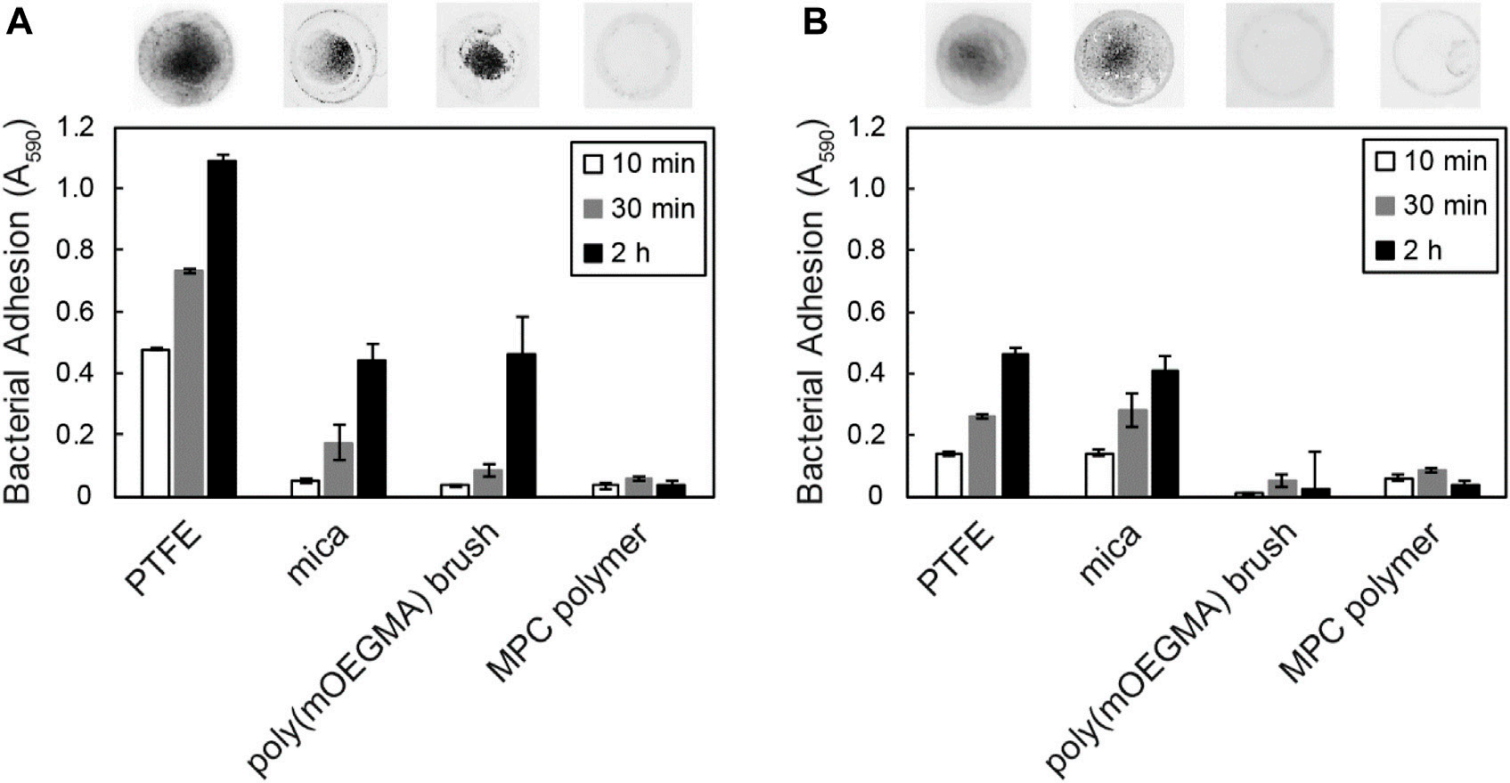
Adhesion of bacterial cells to antiadhesive surfaces. Adhesion of Tol 5 **(A)** and *Bartonella henselae* **(B)** to PTFE, mica, a poly (mOEGMA) brush on glass and MPC polymer-coated glass was assessed by microwell adhesion assays. Data are expressed as the means ± SEMs (*n* = 3). The upper photographs show the quickly adhered cells on the material surfaces after incubation for 2 h.

To investigate whether Tol 5 cells can adhere to various other antiadhesive surfaces in addition to PTFE (Table 1), we measured cell adhesion to mica, poly (oligo (ethylene glycol) methyl ether methacrylate) (poly (mOEGMA)) brush, and 2-methacryloyloxyethyl phosphorylcholine (MPC) polymer surfaces, using the self-made microwells. Mica is a phyllosilicate mineral of aluminum and potassium, and its cleaved surface is atomically flat (de Poel et al., 2014). A poly (mOEGMA) brush is a neutral hydrophilic polymer brush and exerts steric hindrance (Sin et al., 2014). An MPC polymer is a zwitterionic hydrophilic polymer that has a high free water fraction (Ishihara et al., 1998). The surfaces of these materials have been reported to have antiadhesive properties against bacterial cells (Cheng et al., 2007; Zeng et al., 2014; Kameda et al., 2019). During the incubation of bacterial cells on the antiadhesive surfaces for 10 min, Tol 5 cells quickly adhered to the surface of PTFE but not to that of mica, the poly (mOEGMA) brush, or the MPC polymer (Figure 3A). However, after incubation for 2 h, Tol 5 cells also adhered to mica and the poly (mOEGMA) brush surfaces. Nevertheless, Tol 5 cells could hardly adhere to the MPC polymer surface after the 2 h incubation. In contrast, *B. henselae* adhered to the surfaces of PTFE and mica but not to that of the poly (mOEGMA) brush and MPC polymer even after a 2-h incubation (Figure 3B). These results emphasized that Tol 5 cells were the only cells that adhered to the poly (mOEGMA) brush surface and showed that even sticky Tol 5 cells hardly adhered to the MPC polymer surface under these experimental conditions.

### 3.3 Direct measurement of the adhesion force of bacterial cells using AFM

**FIGURE 4:**
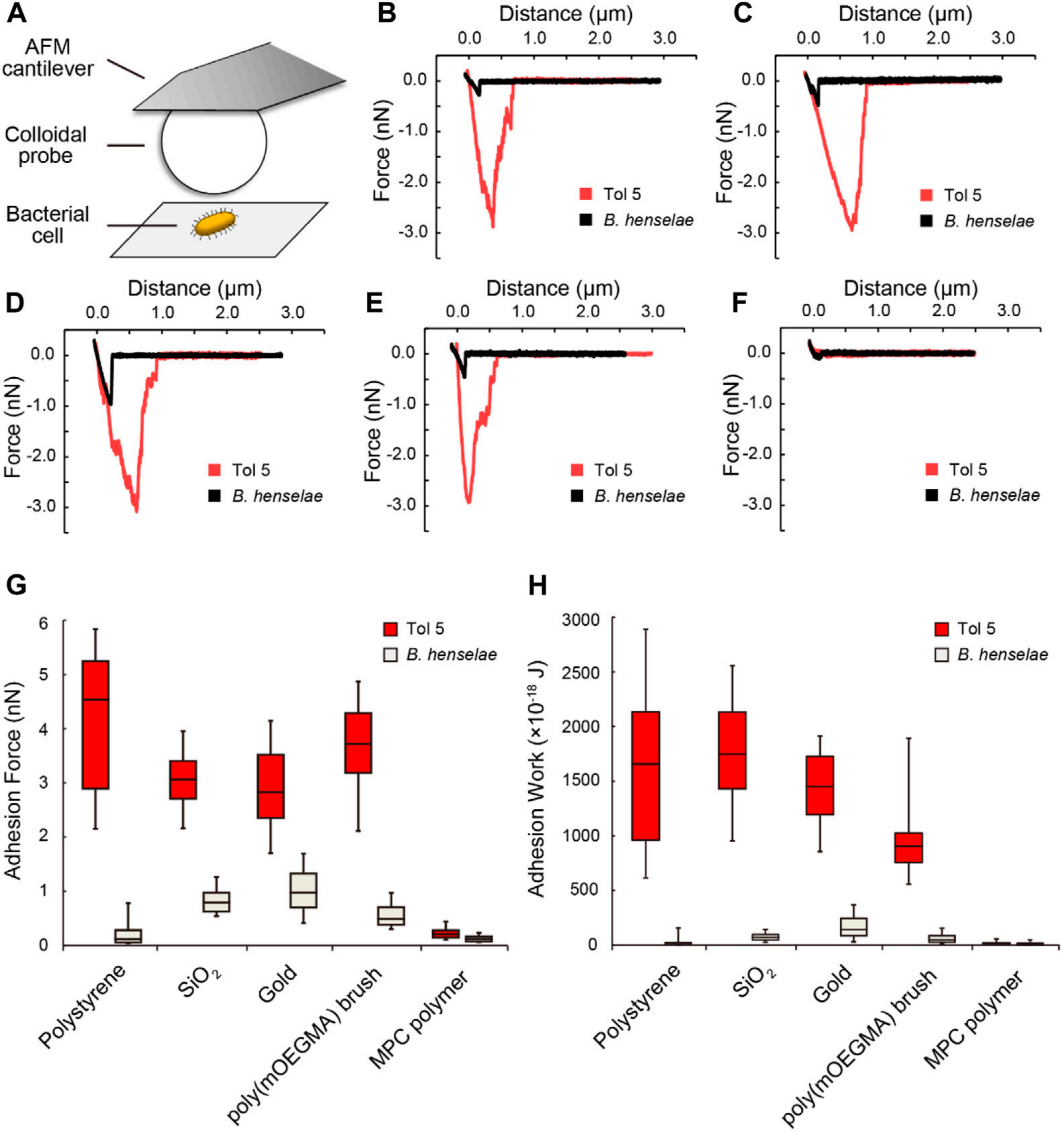
Single-cell force spectroscopy using colloidal probes. **(A)** Schematic illustration of the measurement. **(B–F)** Typical force curves of bacterial cell adhesion to colloidal probes made from various materials: polystyrene **(B)**, SiO_2_ **(C)**, gold **(D)**, a poly (mOEGMA) brush **(E)**, and an MPC polymer **(F)**. **(G, H)** Box and whisker plot of the adhesion force **(G)** and adhesion work **(H)** of bacterial cells adhered to various colloidal probes. The boxes represent the data from the 25th to the 75th percentile, and the whiskers extend to the 10th/90th percentile. The horizontal lines in the box represent the median values. For each pair of bacterial strain and material, at least 60 measurements were performed using cells from at least two independently grown cultures.

In contrast to semiquantitative adhesion assays that measure the amount of cells adhering to microwells by staining, AFM can quantify the strength of adhesion as the adhesion force in Newtons, i.e., the resistance force against peeling (Dufrene, 2015; Elbourne et al., 2019). We previously reported that *Acinetobacter* sp. Tol 5 exhibits an adhesion force of 2 nN to a sharp silicon nitride AFM probe, which is more than 10-fold stronger than that of the *B. henselae* strain (Ishii et al., 2022). To quantify the strength of adhesion of Tol 5 to various materials, including antiadhesive surfaces, compared to that of the *B. henselae* strain, which showed some adhesiveness to PTFE and mica, we measured the adhesion force of a single cell using AFM. To ensure that the adhesion force of a single cell was measured, a single colloidal probe made from PS, SiO_2_, gold, poly (mOEGMA) brush-coated SiO_2_, or MPC polymer-coated SiO_2_ (Table 1) was pressed against the top of a single bacterial cell immobilized on a glass substrate beforehand through a covalent bond between the bottom side of the cell surface and a glass surface, and the force required to pull the probe away from the cell was measured (Figure 4A). Colloids of the same diameter were used for the measurements to equalize the contact area between cells and probes of different materials. This single-cell force spectroscopy demonstrated that Tol 5 exhibited a strong adhesion force over a long distance of 500–1,000 nm (Figures 4B–E). The median of the maximum adhesion forces of Tol 5 to PS, SiO_2_, gold, and even the poly (mOEGMA) brush was 2.5 nN, which was much stronger than that of *B. henselae* (≤1 nN) (Figure 4G). The adhesion work of Tol 5, which is the energy required for detachment and is shown as the peak area of the adhesion force curve, was more than 10 times larger than that of *B. henselae* (Figure 4H). On the other hand, both Tol 5 and *B. henselae* showed only faintly weak adhesion force and small adhesion work when adhered to the MPC polymer (Figures 4F–H). These results highlighted the remarkably strong nonspecific adhesion of Tol 5 to PS, SiO_2_, gold, and even the poly (mOEGMA) brush, but not to the MPC polymer, demonstrating the remarkable antiadhesive property of the MPC polymer.

### 3.4 Behavior of tol 5 cells on the MPC polymer surface under flow

**FIGURE 5:**
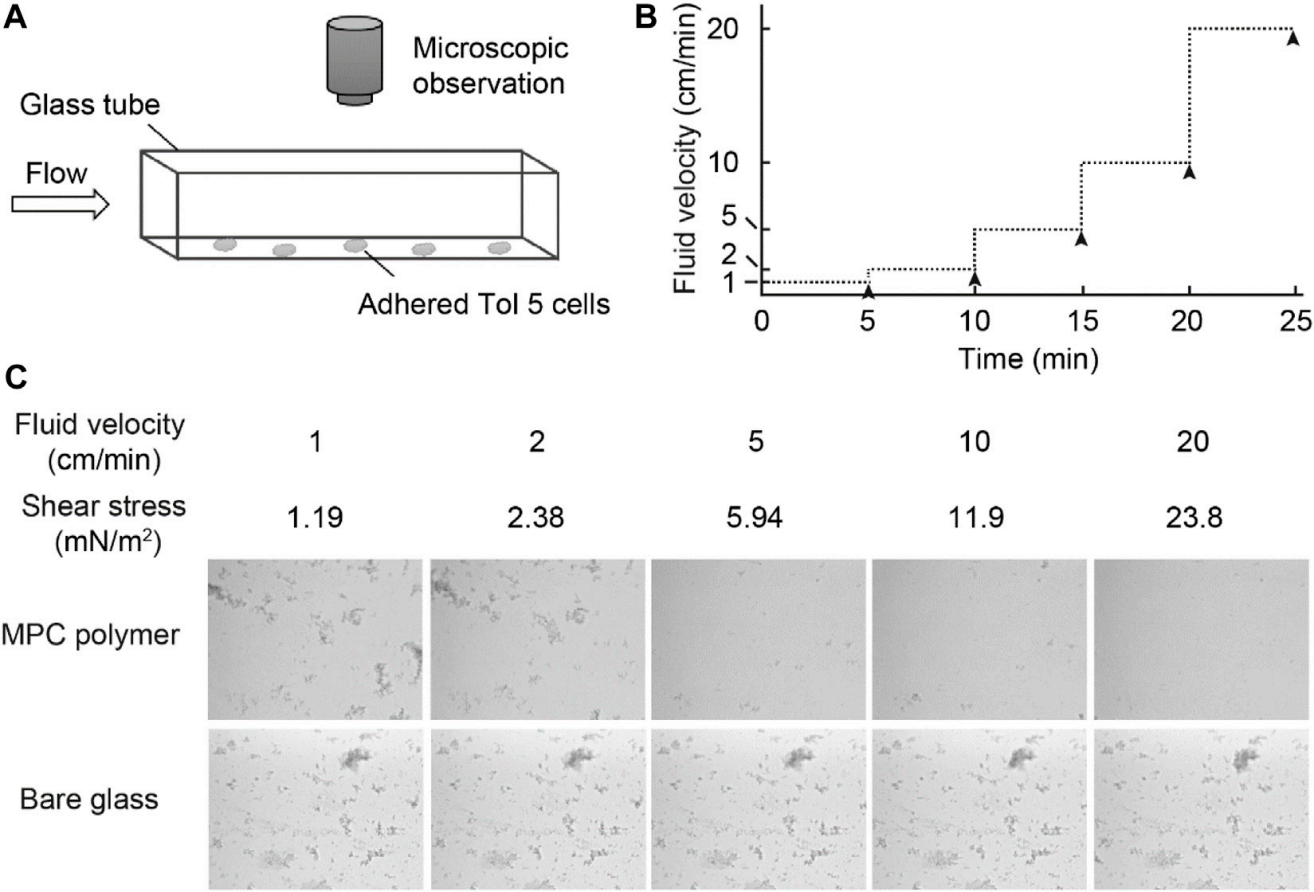
Observation of the behavior of Tol 5 cells adhered to the MPC polymer surface beforehand. **(A)** Schematic representation of the flow chamber system used in this study. **(B)** Transition of the fluid velocity. The fluid velocity was increased stepwise every 5 min. The black arrowheads indicate the times at which snapshots of the inner surface at the bottom of the glass tubes were captured. **(C)** Snapshots captured as described in **(B)**.

To investigate the behavior of Tol 5 cells on the MPC polymer surface in the presence of shear forces, we observed Tol 5 cells on the surface by using a flow chamber system with a square glass tube (Figure 5A) (Furuichi et al., 2018). The glass tube with or without the MPC polymer coating was filled with a Tol 5 cell suspension and incubated for 10 min. Then, the cell suspension was replaced with fresh BS-N buffer by flowing slowly at 1 cm/min for rinsing, and the fluid velocity was increased stepwise, as shown in Figure 5B, while observing the inner surface of the bottom of the glass tube under a microscope. Unexpectedly, Tol 5 cells adhered to the MPC polymer-coated glass as much as to the bare (noncoated) glass under static conditions and remained adhered after rinsing at 1 cm/min (Figure 5C). When the fluid velocity was increased to 2 cm/min, a small fraction of previously adhered cell clumps started to move and slip on the surface (see Supplementary Movie S1), but many cells still resisted detachment after 10 min of flow (Figure 5C, 2 cm/min). At a high fluid velocity of 5 cm/min or more, the Tol 5 cells firmly adhered to the bare glass, whereas the cells attached to the MPC polymer were exfoliated, rolled, and washed away from the surface by shear stress (≥5.94 mN/m^2^) (Figure 5C, ≥5 cm/min; see Supplementary Movie S1).

## 4 Discussion

To date, various antiadhesive materials have been developed on the basis of different repelling mechanisms. Fluoropolymers, including PTFE with a low surface free energy, are widely used not only in cookware but also in labware and medical equipment (Lih et al., 2015). Polymer brushes with a high grafting density have been especially studied as powerful antiadhesive surfaces for cell adhesion based on their hydrophilicity and steric hindrance (Krishnamoorthy et al., 2014). In this study, we examined the adhesion of *Acinetobacter* sp. Tol 5, which exhibits extremely high adhesiveness to various surfaces through AtaA to antiadhesive surfaces. We demonstrate that Tol 5 can adhere to almost any of the so-called antiadhesive surfaces tested. The finding that Tol 5 is able to adhere to these antiadhesive materials shows its remarkably wide range of adhesion preferences. Only a surface coated with MPC polymer was at least partially successful in preventing Tol 5 adhesion. This is the first report on a surface to which Tol 5 cannot adhere.

Although BadA is similar to AtaA in size and its fibrous molecules peritrichously cover the bacterial cells (Muller et al., 2011), the adhesiveness of *B. henselae* to material surfaces was lower than that of Tol 5, and in contrast to Tol 5, *B. henselae* did not adhere to the poly (mOEGMA) brush (Figures 3, 4). Their difference in adhesiveness demonstrated the functional diversity of the TAA family as a result of protein evolution; *B. henselae* is a zoonotic pathogen and has therefore adapted to mammalian surfaces (Thibau et al., 2022), whereas Tol 5 has adapted to environmental and abiotic surfaces. Our study also demonstrates the importance of using a variety of bacteria in the evaluation of antiadhesive surfaces.

Dynamic adhesion experiments using a flow chamber system revealed that Tol 5 cells adhered even to an MPC polymer-coated surface, but their interaction was so weak that the cells could be exfoliated by a weak shear stress (Figure 5). In the static adhesion assays using a microwell (Figure 3), the Tol 5 cells must have been detached from the MPC polymer-coated glass by the washing step. In addition, the exfoliated and rolling cell clumps seemed to incorporate and remove cell clumps that were still adhered owing to the autoagglutinating property of Tol 5 cells (Furuichi et al., 2018), resulting in the self-cleaning of the surface coated with the MPC polymer (see Supplementary Movie S1). This suggested that the autoagglutinating property, which generally promotes adhesion and microcolony formation (Trunk et al., 2018), negatively affects adhesion to the MPC polymer. The high potential of MPC polymers to inhibit adhesion, which can prevent even the adhesion of highly sticky Tol 5, as an antiadhesive material was thus reaffirmed. MPC is a methacrylate monomer with a phosphorylcholine (PC) group, which is a hydrophilic polar head group also present on phospholipids comprising eukaryotic cell membranes (Iwasaki and Ishihara, 2012). The underlying inhibitory mechanism for the adhesion of proteins and cells to MPC polymers is based on their interactions with water molecules: while there are many free water molecules present (Ishihara et al., 1998), only a few bound water molecules are captured by the PC groups of the MPC polymers (Iwasaki and Ishihara, 2012; Nagasawa et al., 2015; Azuma et al., 2017). Tol 5 cells could adhere to the surfaces of the poly (mOEGMA) brush and mica but could interact only very weakly with the surface coated with MPC polymers, despite the similar hydrophilicity of these materials, as shown by the static contact angles of air in water (Supplementary Table S1). This suggested that exchangeable, weakly bound water molecules contribute to the interaction between AtaA and material surfaces.

Bacterial immobilization is an important strategy for the efficient use of whole-cell catalysts. While the nonspecific adhesion of Tol 5 through AtaA is useful for immobilization, it also leads to undesirable adhesion to laboratory tools and equipment during cultivation, collection, and preparation of bacteria. This study showed that Tol 5 cannot adhere to the MPC polymer-coated surface. This finding allows us to handle AtaA-expressing bacteria more efficiently and with fewer complications, as the use of MPC polymer-coated surfaces. This would promote the use of AtaA-expressing bacteria as an immobilized whole-cell catalyst and improve the efficiency of environmentally friendly bioprocesses.

In conclusion, we have investigated the interactions of Tol 5 and several other TAA-expressing pathogenic bacteria with a variety of materials, including antiadhesive surfaces, and revealed their adhesion preferences. Our findings will contribute to the understanding and control of cell adhesion of Tol 5 for immobilized bioprocess applications and other TAA-expressing pathogenic bacteria of medical importance. However, in order to paint a complete picture of the adhesion mechanism of Tol 5 cells, it remains a challenge for future research to clarify the physicochemical factors governing adhesion to materials. It would be helpful to evaluate adhesion to surfaces with controlled physical properties, such as roughness or nanostructure, as well as chemical properties.

## Data Availability

The original contributions presented in the study are included in the article/Supplementary Material, further inquiries can be directed to the corresponding author.
