# Supplementary material for: Adhesion preference of the sticky bacterium Acinetobacter sp. Tol 5
Source: Front Bioeng Biotechnol. 2024 Feb 5;12:1342418. doi: 10.3389/fbioe.2024.1342418 (PMC10875045; doi:10.3389/fbioe.2024.1342418)
Supplement: Supplementary file 1 [file DataSheet1.PDF]

Adhesion preference of the sticky bacterium *Acinetobacter* sp. Tol 5

Shogo Yoshimoto[1], Satoshi Ishii[1], Ayane Kawashiri[1], Taishi Matsushita[2], Dirk Linke[3], Stephan Göttig[4], Volkhard A. J. Kempf[4], Madoka Takai[2], Katsutoshi Hori[1]*.

[1] Department of Biomolecular Engineering, Graduate School of Engineering, Nagoya University, Nagoya, Aichi 464-8603, Japan.

[2] Department of Bioengineering, Graduate School of Engineering, The University of Tokyo, Bunkyo-ku, Tokyo 133-8656, Japan.

[3] Department of Biosciences, University of Oslo, 0316 Oslo, Norway

[4] Institute for Medical Microbiology and Infection Control, University Hospital, Goethe University, Frankfurt 60596, Germany.

* Correspondence:

Katsutoshi Hori

Tel: +81-52-789-3339

Fax: +81-52-789-3218

Email: khori@chembio.nagoya-u.ac.jp

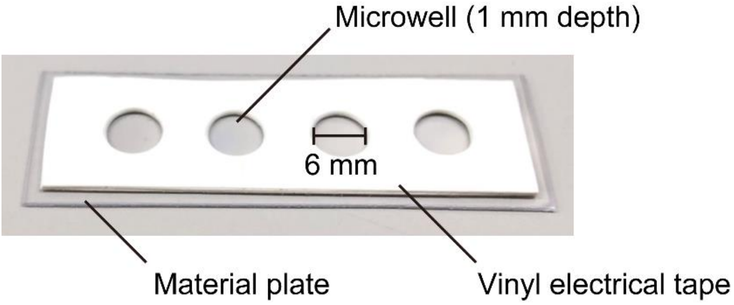

Fig. S1. Preparation of microwells on material surfaces. Four sheets of vinyl electrical tape were stacked, and holes with a diameter of 6 mm were punched into the stacked tape. The punched tape was placed on a material plate.

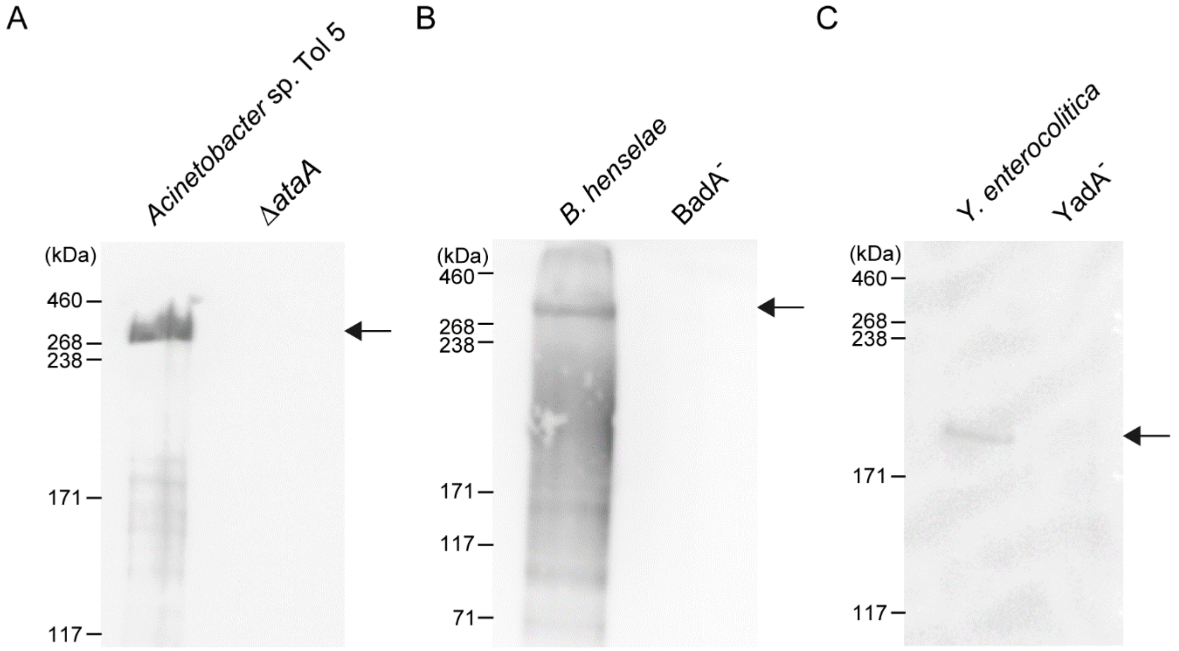

Fig. S2. Production of trimeric autotransporter adhesin (TAA) in each bacterial cell. Cell lysates of *Acinetobacter* sp. Tol 5 (A), *B. henselae* (B), and *Y. enterocolitica* (C) were separated by SDS-PAGE with their TAA-less mutant strains, and each TAA was detected by western blotting. The arrow in each panel indicates the band corresponding to AtaA monomer (A), BadA monomer (B), and YadA trimer (C).

Table S1. Static contact angles (SCA) of air in water of material surfaces. Data are expressed as the mean ± SD (n = 5).

**Material**	**Polystyrene**	**Glass**	**Stainless steel**	**PTFE**	**Mica**	**Poly(mOEGMA) brush**	**MPC polymer**
**SCA [deg]**	99 ± 2	139 ± 3	101 ± 3	86 ± 4	142 ± 3	134 ± 2	154 ± 4

Table S2. Bacterial strains used in this study.

Strain	Description	Reference
Acinetobacter sp. Tol 5	Wild type strain, expressing *ataA*	(Hori et al., 2001)
Acinetobacter sp. Tol 5 Δ*ataA*	*Acinetobacter* sp. Tol 5 4140, Unmarked Δ*ataA* mutant of Tol 5, *ataA*$^{-}$	(Ishikawa and Hori, 2013)
Bartonella henselae	*B. henselae* Marseille, Patient isolate, expressing *badA*	(Drancourt et al., 1996)
Bartonella henselae BadA^{-}	*B. henselae* Marseille transposon mutant, transposon integrated in *badA*, Km^{r}	(Riess et al., 2004)
Yersinia enterocolitica	*Y. enterocolitica* WA-314 serotype O:8, harboring plasmid pYV, expressing *yadA*	(Heesemann and Laufs, 1983)
Yersinia enterocolitica YadA^{-}	*Y. enterocolitica* WA-C, plasmid less derivative of WA-314, *yadA*$^{-}$	(Heesemann and Laufs, 1983)

Movie S1. Detachment of adhered Tol 5 cells from the MPC polymer surface under flow. A glass tube with or without an MPC polymer coating was filled with Tol 5 cell suspension and incubated for 10 min. Then, the cell suspension was replaced with fresh BS-N buffer by flowing slowly at 1 cm/min for 35 min, and the fluid velocity was increased stepwise, as shown in Figure 5B. Live images of the adhesion behavior of Tol 5 cells on the inner surface of the bottom of the glass tube were observed under a microscope.

References

Drancourt, M., Birtles, R., Chaumentin, G., Vandenesch, F., Etienne, J., and Raoult, D. (1996). New serotype of *Bartonella henselae* in endocarditis and cat-scratch disease. *Lancet* 347(8999), 441-443.

Heesemann, J., and Laufs, R. (1983). Construction of a mobilizable *Yersinia enterocolitica* virulence plasmid. *J Bacteriol* 155(2), 761-767.

Hori, K., Yamashita, S., Ishii, S., Kitagawa, M., Tanji, Y., and Unno, H. (2001). Isolation, characterization and application to off-gas treatment of toluene-degrading bacteria. *J Chem Eng Jpn* 34(9), 1120-1126.

Ishikawa, M., and Hori, K. (2013). A new simple method for introducing an unmarked mutation into a large gene of non-competent Gram-negative bacteria by FLP/FRT recombination. *BMC Microbiol* 13, 86.

Riess, T., Andersson, S.G.E., Lupas, A., Schaller, M., Schafer, A., Kyme, P., et al. (2004). *Bartonella* adhesin A mediates a proangiogenic host cell response. *J Exp Med* 200(10), 1267-1278.
